# A 127 kb truncating deletion of *PGRMC1* is a novel cause of X-linked isolated paediatric cataract

**DOI:** 10.1038/s41431-021-00889-8

**Published:** 2021-04-19

**Authors:** Johanna L. Jones, Mark A. Corbett, Elise Yeaman, Duran Zhao, Jozef Gecz, Robert J. Gasperini, Jac C. Charlesworth, David A. Mackey, James E. Elder, Jamie E. Craig, Kathryn P. Burdon

**Affiliations:** 1 Menzies Institute for Medical Research, University of Tasmania, Hobart, TAS Australia; 2 Adelaide Medical School, Robinson Research Institute, University of Adelaide, Adelaide, SA Australia; 3 School of Medicine, University of Tasmania, Hobart, TAS Australia; 4 Centre for Ophthalmology and Visual Science, University of Western Australia, Lions Eye Institute, Perth, WA Australia; 5 Department of Paediatrics, University of Melbourne, Melbourne, VIC Australia; 6 Department of Ophthalmology, Flinders University, Bedford Park, SA Australia

**Keywords:** Genetic linkage study, Next-generation sequencing

## Abstract

Inherited paediatric cataract is a rare Mendelian disease that results in visual impairment or blindness due to a clouding of the eye’s crystalline lens. Here we report an Australian family with isolated paediatric cataract, which we had previously mapped to Xq24. Linkage at Xq24–25 (LOD = 2.53) was confirmed, and the region refined with a denser marker map. In addition, two autosomal regions with suggestive evidence of linkage were observed. A segregating 127 kb deletion (chrX:g.118373226_118500408del) in the Xq24–25 linkage region was identified from whole-genome sequencing data. This deletion completely removed a commonly deleted long non-coding RNA gene *LOC101928336* and truncated the protein coding progesterone receptor membrane component 1 (*PGRMC1*) gene following exon 1. A literature search revealed a report of two unrelated males with non-syndromic intellectual disability, as well as congenital cataract, who had contiguous gene deletions that accounted for their intellectual disability but also disrupted the *PGRMC1* gene. A morpholino-induced *pgrmc1* knockdown in a zebrafish model produced significant cataract formation, supporting a role for PGRMC1 in lens development and cataract formation. We hypothesise that the loss of PGRMC1 causes cataract through disrupted PGRMC1-CYP51A1 protein–protein interactions and altered cholesterol biosynthesis. The cause of paediatric cataract in this family is the truncating deletion of *PGRMC1*, which we report as a novel cataract gene.

## Introduction

Cataracts are an opacification of the lens of the eye, a normally transparent structure responsible for transmitting an undistorted and focused image onto the retina. Cataracts are classified according to their age of onset. Childhood onset cataracts (congenital or paediatric) are considered to be a rare disease, with an estimated global prevalence of 4.24 per 10,000 [1] and 2.2 per 10,000 births [2] for the Australian population specifically. Paediatric cataracts are known to have genetic basis in up to 25% of cases [3–5].

Paediatric cataract is typically a highly penetrant monogenic disease; autosomal dominant, autosomal recessive and X-linked inheritance patterns have all been reported [6]. To date, at least 52 genes and loci have been reported to cause isolated paediatric cataract [7] with two of these located on the X chromosome. Variants in the *NHS* gene (MIM: 302200) can cause isolated X-linked cataract [8–10], although they more commonly cause Nance–Horan syndrome (MIM: 302350), characterised by congenital cataracts, dental anomalies, dysmorphic features and, in some cases, mental retardation [11]. Other X-linked syndromic paediatric cataract genes include *ARSE* (MIM: 300180), *BCOR* (MIM: 300485), *NDP* (MIM: 300658), *EBP* (MIM: 300205), *UBE2A* (MIM: 312180), *OCRL* (MIM: 300535) and loci causing: diffuse leiomyomatosis with Alport syndrome (DL-ATS [MIM: 308940]), contiguous ABCD1/DXS1375E deletion syndrome (CADDS [MIM: 300475]) and CASM syndrome (MIM: 300619). The only other known X-linked locus for isolated cataract is a region on Xq24 that was previously reported by our group in a family with isolated paediatric cataract [12]. Here we describe the identification of the causative variant in this family.

**Fig. 1:**
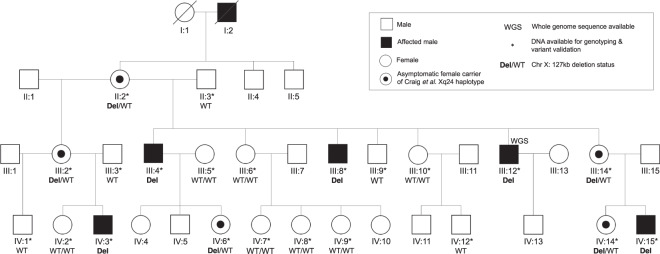
Pedigree of family CRVEEH66 and candidate variant status. The previously studied CRVEEH66 family [12]. An ophthalmologist confirmed cataract affection status and no clinically significant lens opacity was observed in females. Segregation of 127 kb deletion (Del), at Xq24, is displayed. All affected males are hemizygous for the deletion and obligate female carriers are heterozygous for the variant (Del/WT) as is female IV:14.

A full description of the three-generation family (CRVEEH66, Fig. 1) and clinical descriptions of affected individuals have been previously reported [12]. Briefly, the five affected males show a lamellar or nuclear cataract with onset between 4 and 22 years of age with slow disease progression until they require surgery in late teens or adulthood. A range of other variable ocular and non-ocular features was also recorded, including astigmatism, glaucoma, dental crowding and pyramidal incisors, high arched palate and facial dysmorphism. It is not known if these features are related to the cataract or are coincidental. Obligate female carriers display no clinically significant lens opacification or other notable features. Linkage analysis using microsatellite markers was previously performed on chromosome X and a 11.5 Mb region of linkage was identified (max LOD = 2.53) between markers DXS8055 and DXS8009, at the Xq24 locus [12]. To identify the causative variant in this family, we used a SNP array to fine map the Xq24 linkage region and assess the possibility of autosomal inheritance with reduced expressivity in females. We then used whole-genome sequencing to identify candidate variants and a zebrafish model to assess the role of the candidate gene in cataract formation.

## Materials and methods

### Study participants and control cohorts

Recruitment, clinical examination and DNA extraction of the 22 individuals from this family have been previously described [12]. All participants gave written informed consent and protocols were approved by the Tasmania Health and Medical Human Research Ethics Committee, the Southern Adelaide Clinical Human Research Ethics Committee and Royal Victorian Eye and Ear Hospital Human Research Ethics Committee. The frequency of segregating structural variants was assessed in Australian population controls randomly selected from the Blue Mountains Eye Study (*n* = 70) [13].

### Genotyping and linkage analysis

Genome-wide genotyping of all available family members (*n* = 22) was performed using a HumanOmniExpress-24 v1.1 SNP array (Illumina) and imaged using an Illumina HiScan System. GenomeStudio™ 2011.1 software (Illumina) with the appropriate manifest and cluster files was used to perform quality control for the genotyping data. SNPs were excluded if they failed to meet the recommended 0.15 GenCall threshold (Illumina), had a call frequency less than 100%, had ambiguous genotype clusters or if males had a heterozygous chromosome X genotype. Data were exported to PLINK format using the PLINK Input Plugin v2.1.1. Data were restricted to a set of independent SNPs for autosomal linkage analysis based on linkage disequilibrium pruning in PLINK v1.9 [14] on an independent population of 1585 Caucasians also genotyped on HumanOmniExpress SNP arrays (Illumina) as part of a previous study [15]. Files were converted from PLINK PED format to Merlin format using Mega2 v4.9.2 [16, 17]. Mendelian inconsistent autosomal SNPs were removed, as well as those on the X chromosome, which were identified using the ‘minx’ and error detection functions in Merlin [18].

Parametric multipoint linkage analysis was performed using Merlin v1.1.2 [18, 19]. Analysis was run with a dominant disease model; with a disease allele frequency of 0.0001 and locus penetrance of 0.0001, 0.90 and 1.0 for genotypes 0/0, 0/1 and 1/1, respectively. Males were set as either affected or unaffected based on their clinical phenotype. Females were set as unknown regardless of known carrier status and married-in family members were set to unaffected. To fit the 24-bit upper limit in Merlin, an uninformative branch of the pedigree containing individual III:6 and her offspring IV:7, IV:8 and IV:9 was excluded from the linkage analysis. The identification of critical recombination events defining the disease haplotype was performed using Merlin haplotype analysis.

### Whole-genome sequencing and variant filtering

Whole-genome sequencing of an affected male (III:12) was performed using the Complete Genomics pipeline, CGA™Tools version 2.4.0.37 [20], and alignment to human reference genome hg19. Variants with quality = ‘pass’ were filtered by variant type; copy number variants (CNV) filtered to ‘knownCNV’ = 0 and structural variants filtered ≤ 0.04 in ‘FrequencyBaselineGenomeSet’ were all obtained from the CGA™Tools software outputs. SNV and Indels were annotated using ANNOVAR [21] and filtered to functional ‘exonic’ or ‘splicing’ variants and MAF ≤ 0.001 in ExACv1, ≤0.005 in Exome Variant Server and UK10K control cohorts and ≤0.01 in all other databases. All variants meeting the filtering criteria from within the linkage regions were validated in the proband and assessed for segregation in the family. Validated variants have been submitted to ClinVar (https://www.ncbi.nlm.nih.gov/clinvar/) with accession numbers reported.

### Variant validation and candidate gene analysis

PCR, gel electrophoresis and Sanger sequencing were used to validate each variant in individual III:12, assess segregation in the remainder of the family and to determine the frequency in the Australian control cohorts. Primers were designed, using NCBI PrimerBlast [22] (Table S1). Genomic DNA was amplified using MyTaq^™^ HS mix (Bioline) according to the manufacturer’s instructions and visualised using a 1.5–2% (w/v) agarose gel electrophoresis. PCR products were purified using Agencourt AMPure XP beads (Beckman Coulter) according to manufacturer’s instructions.

Sequencing reactions were performed using a BigDye® Terminator v3.1 Cycle Sequencing Kit (Applied Biosystems) before reactions were purified using Agencourt CleanSEQ beads (Beckman Coulter). Denatured sequencing products were electrophoresed on an ABI 3500 Genetic Analyser (Life Technologies) with POP7 polymer (Life Technologies) and a 50 cm eight capillary array.

Candidate genes were assessed using bioinformatic tools, publicly available databases and a comprehensive literature search, for evidence to support or negate a role in lens biology and cataractogenesis.

### Zebrafish and embryo maintenance

All animal procedures used in this study were approved by the University of Tasmania Animal Ethics Committee in accordance with the Australian National Health and Medical Research Council Code of Practice for the Care and Use of Animals for Scientific Purposes. Adult zebrafish were maintained on a 14/10-h light/dark cycle in standard conditions [23]. Embryos were obtained from natural spawning events of an available Gal4^s1020t^/UAS:mCherry transgenic line [24, 25] previously gifted by Dr. Ethan Scott (University of Queensland). All embryos destined for lens imaging were raised in 1xPTU (200 µM, 1-phenyl-2-thiourea (Sigma-Aldrich)) from 24 h post fertilisation, to suppress pigment formation.

### Zebrafish *pgrmc1* gene expression

Expression was confirmed for progesterone receptor membrane component 1 (*pgrmc1*, ENSDART00000076328.5), the zebrafish orthologue of human *PGRMC1*. Zebrafish larvae were collected in RNA*later* RNA Stabilisation Reagent (Qiagen) at 3–5 days post fertilisation from natural spawning events. RNA was extracted using a RNeasy® Plus Mini Kit (Qiagen) according to manufacturer’s instructions and reverse transcribed to cDNA using an Invitrogen SuperScript® III First Strand synthesis system (Thermo Fisher Scientific). Standard PCR was used to amplify the cDNA with primers designs for the desired transcript (Table S1) prior to visualisation on 2% w/v agarose gel and confirmed using Sanger sequencing.

### Morpholinos

Two independent *pgrmc1* targeting morpholino antisense oligonucleotides (MO; GeneTools LLC) were used for gene knockdown experiments in zebrafish. This included a translation start site blocking morpholino (*pgrmc1*_MO1*;* 5′GCTCGACTGCTTCTTCAGCCATTTC 3′) that has previously been reported to knockdown zebrafish *pgrmc1* [26, 27] and an exon 1/intron 1 splice site targeting morpholino (*pgrmc1*_MO2; 5′ATATTTAAGTTGATACCTGGACCGT 3′).

A standard control morpholino targeting the human beta-globin intronic sequence containing a variant known to cause beta-thalassaemia was used as a negative control (5′ CCTCTTACCTCAGTTACAATTTATA 3′, GeneTools LLC). A morpholino targeting the aquaporin gene (*aqp0a*_MO; 5′AACTCCCACATGGCTGCAAAAAGTC 3′) and previously shown to cause cataract in zebrafish [28] was used as a positive control. A 1 mM stock concentration, or ~8 ng/nL, of each morpholino was created using distilled water according to the manufacturer’s instructions. Dosage optimisation was performed to obtain the maximum dose prior to the formation of gross morphological defects in the larvae. Calibrated 1 nL microinjections of standard control MO (at 8 ng/nL, 8 ng dose), *pgrmc1_*MO1 (optimised at 2 ng/nL, 2 ng dose) and *pgrmc1_*MO2 (optimised for a 1.5 nL injection to produce a 12 ng dose) were performed into one to two cell stage embryos of Gal4^s1020t^/UAS:mCherry transgenic zebrafish. Matched experimental and control larvae were all collected in RNAlater following imaging. Whole RNA was extracted for assessment of *pgrmc1_*MO2 splice-altering efficiency with RT-PCR and efficiency of *pgrmc1*_MO1 was assumed based on the previous work that had been performed [26, 27].

### Imaging and analysis

Zebrafish lenses were assessed for cataract at 4 days post fertilisation. Larvae were anaesthetised in tricaine methanesulfonate (300 mg/L, MS222 (Glentham Life Sciences), pH 7.0) and whole mounted in 0.5% w/v agarose and imaged using differential interference contrast illumination, under ×40 magnification, using an inverted microscope (Nikon Eclipse Ti-E) equipped with an s-CMOS camera, Zyla 4.2 plus s-CMOS camera (Zyla 4.2, Andor) and NIS-Elements AR acquisition software (Nikon).

Lens images from all experiments were collated, blinded and viewed with ImageJ [29] for cataract assessment by two independent researchers. Any divergent calls were resolved by a third researcher to obtain a result. Images were assessed for structural and organisational defects using a rubric of images. *P* values were calculated using a Fisher’s Exact test and experimental replicates were performed to confirm results. A post hoc power analysis (G*Power 3.1.9.4 [30]) from the *aqp0a* positive control experiments was used to determine that a sample size of >93 total embryos was required to achieve 80% power to detect an effect of size of 0.29, which is equivalent to the cataract rate of 16% observed with this morpholino in our laboratory.

## Results

### Linkage at Xq24–25 confirmed in the studied family

**Fig. 2:**
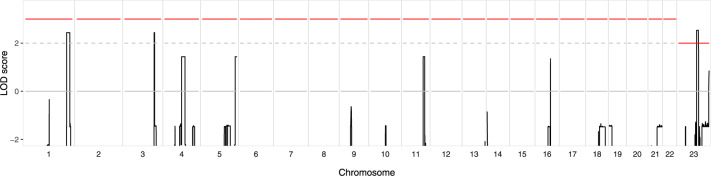
CRVEEH66 genome-wide parametric linkage analysis results. Multipoint LOD scores (*y-*axis), indicating evidence for linkage, are displayed across the genome (*x*-axis). A linkage peak on chromosome X (Chr. 23), between markers rs2428312 and rs7887767, reaches significance with a maximum LOD score of 2.53. Regions on both chromosome 1 (rs696859–rs1316440) and chromosome 3 (rs4527385–rs4857688) reached maximum LOD scores of 2.44, with autosomal LOD scores exceeding two suggestive evidence of linkage (dashed line). The cut-off value for significance is indicated by the red line.

Genome-wide multipoint parametric linkage analysis was performed, and significant evidence of linkage was observed at Xq24–25 with a maximum LOD of 2.53 between markers rs2428312 and rs7887767 (Fig. 2). The increased marker density of the SNP array compared to the microsatellites used by Craig et al. [12] fine-mapped the Xq24–25 region to 6.8 Mb, reducing the size of the area from the 11.5 Mb region previously identified in this family. Two autosomal regions were suggestive of linkage, both obtaining a maximum LOD of 2.44, which were observed between markers rs696859 and rs1316440 at 1q42.2–q43, and at 3q26.31–q26.32 between markers rs4527385 and rs4857688 (Fig. 2).

### Identification of segregating 127 kb deletion at Xq24 as the causative variant

A whole-genome sequence of affected individual III:12 was used to identify candidate variants within the linkage regions. Sequence coverage was at a depth of 30 reads or more for over 80% of the mappable regions in hg19 genome build (Fig. S1). Median sequence coverage of the linkage interval on chromosome X was 21 reads. All variants meeting the filtering criteria from within the linkage regions were validated in the proband and assessed for segregation with disease in the family (Table 1).

**Table 1:** Rare coding or structural variants identified within CRVEEH66 linkage regions that were assessed for validation and segregation.

Structural variants							
Linkage region	Variant type	Genomic position (hg19)	Gene/s	Val	Seg	Freq. in controls	Database ID
Xq24–25	Deletion (127 kb)	NC_000023.10:g.118373226_118500408del	*PGRMC1 LOC101928336*	Yes	Yes	0%	ClinVar:SCV001450779
3q26.31–q26.32	Tandem duplication (364 bp)	NC_000003.11:g.176236711_176237056dup	(intergenic)	Yes	Yes	7.14%	ClinVar:SCV001450780 dbVar:nsv4651094
1q24.2–43	Inversion (104 bp)	NC_000001.10:g.237566103_237566207inv	*RYR2*	No	–	–	–
1q24.2–43	Complex distal duplication (363 bp)	NC_000001.10:g.240116680_240116681ins[GTGTGTGAG;240116016_240116379inv;CCAG]	(intergenic)	Yes	No	–	ClinVar:SCV001450781 gnomAD_SV_v2.1:CPX_1_410
SNPs and indels							
Linkage region	Gene	Genomic position (hg19)	Nucleotide change	Protein change	Val	Seg	Database ID
1q24.2–43	*ERO1B* NM_019891.4	NC_000001.10:g.236399100 G > A	c.662 C > T	p.(Ala221Val)	Yes	Yes	ClinVar:SCV001450782 dbSNP:rs966390978
1q24.2–43	*RYR2* NM_001035.3	NC_000001.10:g.237656318_237656318insC	c.1893_1894insC	p.(Leu631fs)	No	–	–
3q26.31–q26.32	*NAALADL2* NM_207015.3	NC_000003.11:g.175189416_175189417delinsA	c.1534-11_1534-10delinsA	–	Yes	No	ClinVar:SCV001450783 dbSNP:rs1553904048

Coding and protein changes are specific to gene accession reported; Val, validation of variant in proband III:12; Seg, segregation of variant in additional family members; ‘–’, not required. Freq in controls; assessment of structural variants in available DNA control samples (*n* = 70) Blue Mountains Eye Study cohort [13].

Evaluation of autosomal variants identified two segregating candidates. A tandem-duplication event in the 3q26.31–q26.32 locus was commonly observed in controls and was therefore excluded from further analysis. An *ERO1B* c.662 C > T variant was identified in the 1q24.2–43 linkage region; however, although the p.Ala221Val amino acid change was highly conserved, valine is observed at this position in the chimpanzee, our closest relative (Pan troglodytes). The amino acid change was also predicted to be tolerated and benign by SIFT (score = 0.06) and PolyPhen2 (score = 0.061), respectively [31, 32]. Additionally, the *ERO1B* variant had an unlikely pattern of inheritance with eight asymptomatic female variant carriers in addition to all the affected males (obligate carriers II:2, III:2 and III:14, and non-obligate carriers III:6, III:10, IV:2, IV:7 and IV:8). Given the lack of predicted pathogenicity and the unlikely inheritance pattern, this variant was not considered a strong candidate.

**Fig. 3:**
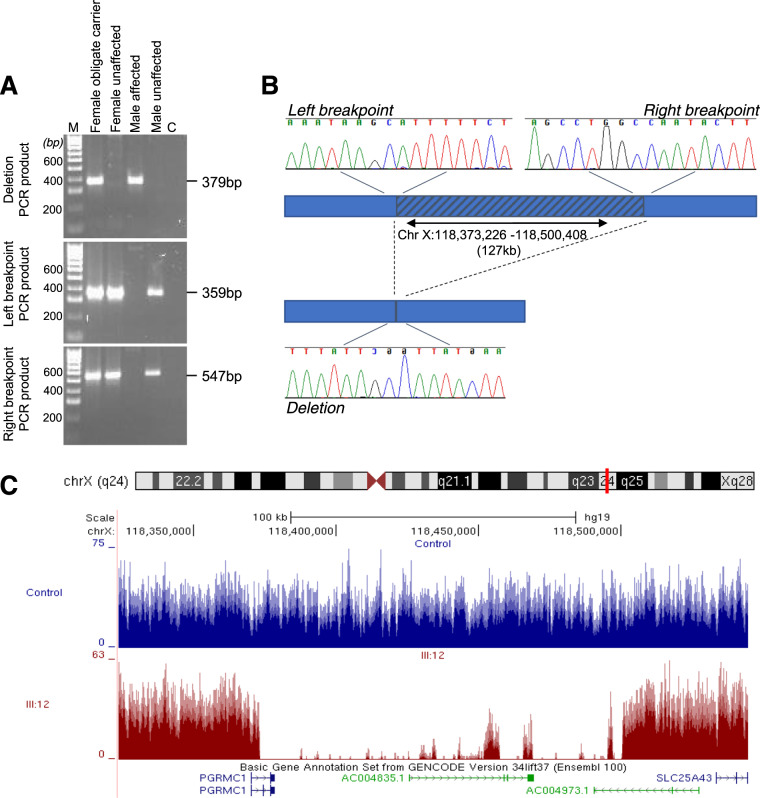
CRVEEH66 127 kb Xq24 deletion variant validation. **A** Agarose gel electrophoresis results of representative samples for PCR products: deletion, left breakpoint, and right breakpoint. Heterozygous female obligate carrier and hemizygous affected male both possess the deletion amplicon. Unaffected female and male samples display wild-type results, with amplicons for breakpoints only. M; 100 bp ladder, C; PCR no template control. **B** Diagrammatic representation of the 127 kb deletion, indicated by the hatched region. Sequencing chromatograms at the critical breakpoints show consensus with the related halves of the deletion sequence. Deletion PCR fragment displayed from sequencing with the reverse primer. **C** UCSC genome browser displays the whole-genome sequence coverage from III:12 in dark red compared to an unrelated control sample in blue focused on the region surrounding the *PGRMC1* gene. The coverage peaks within the deletion region (as indicated by the marked drop in coverage) in individual III:12 correspond with repeat regions in the genome (data not shown) and likely represent spurious mapping.

At Xq24, the only segregating variant identified was a 127 kb deletion (chrX:g.118373226_118500408del). Exact breakpoints were determined using a combination of agarose gel electrophoresis and Sanger sequencing. The deletion is also clearly detectable on a plot of mapped reads from the whole-genome sequence (Fig. 3). The segregation of this 127 kb deletion is consistent with X-linked inheritance in the family and was observed in all affected males (III:4, III:8, III:12, IV:3 and IV:15), all asymptomatic obligate carrier females (II:2, III:2, III:14 and IV:6) and female (IV:14; Fig. 1). This variant completely removes an uncharacterised long non-coding RNA gene *LOC101928336* and truncates the *PGRMC1* gene following exon 1.

### Evaluation of candidate genes within the segregating deletion identified PGRMC1

Deletion of the uncharacterised long non-coding RNA gene, *LOC101928336*, is unlikely to be disease causing. No gene expression was observed in lens epithelial cells in the FANTOM5 expression atlas [33] and the gene is commonly deleted in healthy individuals with a frequency of 0.52% in dbVar (variant gssvL137215, Database of Genomic Variants [34]) and a frequency of 5.2% in Southeast Asian Malays [35]. Expression of *PGRMC1* was observed in lens epithelial cells (FANTOM5 [33, 36]), and the gene is rarely deleted with only a single comparable deletion observed in publicly available data sets (DEL_X_189189, gnomAD structural variant v2.1 [37]). The truncating deletion disrupts PGRMC1’s functional cytochrome b5-like haem-binding domain. We therefore hypothesised that the truncation of the *PGRMC1* gene is a loss of function variant and causative of the cataract phenotype observed in the CRVEEH66 family.

The *PGRMC1* gene has not previously been reported to cause cataract, but a literature search identified a study by Vandewalle et al. [38] that mapped X-linked intellectual disability (NS-ID) to the adjacent *SLC25A5* gene. They report two unrelated male patients, with deletions also impacting *PGRMC1*, who had congenital cataract in addition to intellectual disability. In that study, ‘Patient B’ had a 295 kb deletion (chrX:g.118353178_118648846del) and ‘Patient C’ had a 233 kb deletion (chrX:g.118373287_118606088del) that completely remove and truncate the *PGRMC1* gene following exon 1, respectively [38] (Fig. S2). These two patients, in addition to the family with congenital cataracts we studied, provide strong evidence that loss of function variants in *PGRMC1* cause X-linked paediatric cataract.

### Morpholino-induced *pgrmc1* knockdown results in cataract formation in zebrafish

**Fig. 4:**
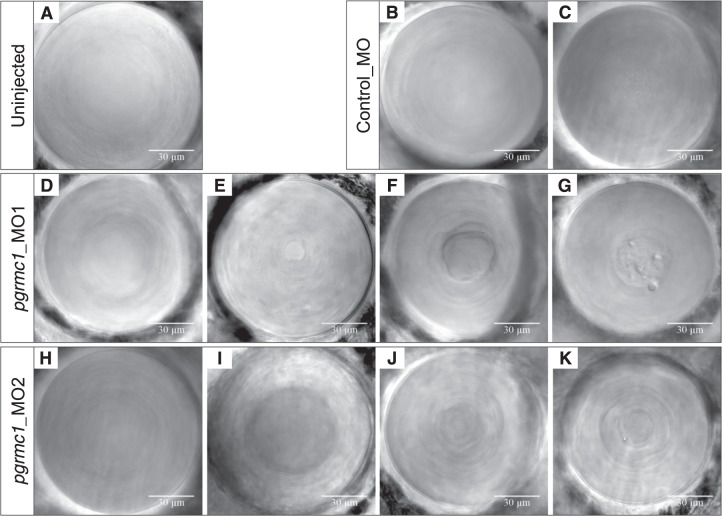
Zebrafish *pgrmc1* morpholino-induced knockdown lens phenotypes. Representative DIC lens images from *pgrmc1* morpholino experiments. Wild-type Gal4^s1020t^/UAS:mCherry transgenic zebrafish display healthy lenses (**A**). Control fish injected with standard control morpholino display healthy lens (**B**) and minor cataract with very fine nuclear pitting (**C**). Fish injected with *pgrmc1_*MO1 (2 ng) morpholino displayed both healthy lenses (**D**) or cataracts of variable severity; minor nuclear central mass (**E**), mild nuclear central mass with fibre cell disorganisation (**F**) and moderate/severe nuclear density with pitting (**G**). Fish injected with *pgrmc1_*MO2 (12 ng) morpholino displayed mainly healthy lenses (**H**) or subtle cataracts of variable severity; minor distinct central lens density (**I**), minor nuclear fibre cell disorganisation (**J**) and moderate nuclear fibre cell disorganisation with minor pitting (**K**).

A zebrafish model was used to further confirm the effect of loss of *PGRMC1* on cataract formation in vivo. Protein alignment between human and zebrafish PGRMC1 orthologs indicates 75% residue identity. Wild-type gene expression was confirmed in zebrafish whole larvae (Fig. S3). Morpholino-induced knockdown of zebrafish *pgrmc1* produced larvae with cataracts of varying degrees of severity. Knockdown using the translation-blocking *pgrmc1_*MO1 morpholino resulted in cataract formation in up to 50% of larvae compared to controls (*P* < 0.0001, Table 2). In comparison to the lens phenotype of uninjected (Fig. 4A) and control injected (Fig. 4B, C) larvae, the *pgrmc1_*MO1 injected larvae displayed nuclear cataract phenotypes of varying severity (Fig. 4D–G). Similarly, subtle nuclear cataracts were observed with a second morpholino, *pgrmc1*_MO2 (Fig. 4H–K); however, there was no statistical difference in the rate of cataract formation observed between *pgrmc1*_MO2-injected larvae and controls (Table 2). RT-PCR was used to assess the splicing mode of action of exon 1/intron 1 boundary targeting *pgrmc1*_MO2. *Pgrmc1*_MO2-treated larvae displayed a retention of intron 1 within the *pgrmc1* transcript. However, they also displayed normally processed *pgrmc1* transcripts that would be expected to be translated into functional protein (Fig. S3).

**Table 2:** Rates of cataract in *pgrmc1* MO versus control MO injected zebrafish larvae at 4 days post fertilisation.

	No. (%) fish		
	Cataract	No cataract	*P* value, OR (95% CI)
Run 1			
* pgrmc1*_MO1 2 ng	12 (50%)	12	<0.0001, –^a^
Control MO	0 (0%)	39	
Run 2			
* pgrmc1*_MO1 2 ng	27 (45%)	33	<0.0001, –^a^
Control MO	0 (0%)	37	
Run 1			
* pgrmc1*_MO2 12 ng	2 (1.9%)	101	0.504, –^a^
Control MO	0 (0%)	82	
Run 2			
* pgrmc1*_MO2 12 ng	7 (4.1%)	164	0.584, 0.7 (0.2–2.1)
Control MO	7 (5.7%)	115	

Statistical significance of experiment group in comparison to control calculated using Fisher’s exact test.

–^a^Odds ratio (OR) unable to be calculated due to ‘0’ value.

## Discussion

The *PGRMC1* truncating 127 kb deletion is a novel cause of the X-linked isolated paediatric cataracts in this Australian family. This variant is located in the reported Xq24 linkage region for this family [12]. The deletion segregates in the family, with all affected males hemizygous for the deletion and all obligate female carriers heterozygous for the variant. Cataract formation following morpholino-induced gene knockdown in zebrafish and an additional report of two other unrelated males with non-syndromic intellectual disability and *PGRMC1*-disrupting deletions and congenital cataracts [38] support this as a novel gene involved in cataractogenesis.

PGRMC1 is member of a group of proteins that contain a homologous CYP5-like haem/steroid binding domain. PGRMC1 is a multifunctional protein that is active in numerous biological processes that could account for cataractogenesis. However, with interactions to known cataract-associated genes involved in cholesterol biosynthesis, we hypothesise that the loss of PGRMC1, and a disruption in this pathway, is causing the cataract phenotype observed in our family.

PGRMC1 forms stable complexes with the CYP51A1 enzyme [39, 40]. Also known as lanosterol 14-alpha demethylase, CYP51A1 performs a critical role in the synthesis of cholesterol through oxidative removal of two methyl groups from the lanosterol intermediate [41]. *PGRMC1* knockdown, using RNAi knockdown in HEK293 cells, causes reduced CYP51A1 activity and results in lanosterol accumulation [39]. In humans, genetic variants in the *CYP51A1* gene (MIM: 601637) have previously been reported to cause congenital cataract with autosomal recessive inheritance [42–44]. PGRMC1 also has reported protein interactions with FDFT1 (squalene synthase, upstream enzyme in cholesterol synthesis pathway) [40], with *FDFT1* (MIM: 184420) and *LSS* (lanosterol synthase [MIM: 600909]) variants attributed to the phenotype of the Shumiya cataract rat model [45]. In humans, variants in the *LSS* gene (an enzyme that catalyses the prior step in the cholesterol pathway to CYP51A1) have also been reported to cause autosomal recessive congenital cataract [46]. These autosomal recessive modes of inheritance are the autosomal equivalent of X-linked disorders, as observed in our family.

Lens fibre cell plasma membranes have an extremely high cholesterol content, with regions of pure cholesterol (lipid rafts) that assist in maintaining cholesterol saturation in the surrounding phospholipid bilayer [47]. *PGRMC1* knockdown and overexpression in vitro have been shown to result in significantly lower and higher levels of lipid rafts, respectively, which is thought to be working through PGRMC1–FDFT1 interactions [40]. In our family, we see the late onset of a clinically significant cataract in males who did not require surgery until their late teens or adulthood [12]. It is hypothesised that a deficiency in cholesterol loading into cell membranes may be particularly problematic in the lens cells. If long-lived lens fibre cells naturally require a very high cholesterol level, insufficient loading and no capacity to address any deficiency following the loss of organelles during maturation could result in cataract development over time.

A significant rate of cataract formation was observed in zebrafish with the translation-blocking morpholino. Although this was not supported by the splice-altering morpholino, its reduced efficiency and lack of cataract formation is supportive of the inheritance pattern observed in the family and other recessive cataract disease genes in the cholesterol biosynthesis pathway. A stable *pgrmc1* knockout zebrafish would be required to definitively confirm gene loss and cataract formation and assess the underlying biology. Lipid profiles are currently unavailable in family members but would provide biochemical confirmation.

We have utilised genome-wide parametric linkage analysis and whole-genome sequencing to identify a causative variant in an Australian family with a previously reported novel isolated paediatric cataract locus at Xq24 [12]. A segregating 127 kb *PGRMC1* truncating deletion was identified at the Xq24 locus. Cataract formation was evident following *pgrmc1* knockdown in a zebrafish model. PGRMC1-disrupting deletions and congenital cataract phenotypes have been observed in two unrelated males. These data strongly suggest that the 127 kb deletion is causative of disease in our family and *PGRMC1* is a novel isolated paediatric cataract gene.

## Supplementary information

Supplementary material
