# Supplementary material for: A 127 kb truncating deletion of PGRMC1 is a novel cause of X-linked isolated paediatric cataract
Source: Eur J Hum Genet. 2021 Apr 19;29(8):1206–15. doi: 10.1038/s41431-021-00889-8 (PMC8385038; doi:10.1038/s41431-021-00889-8)
Supplement: Supplementary file 1 — Supplementary material [file 41431_2021_889_MOESM1_ESM.pdf]

Table S1 – Primer sequences and annealing conditions

Gene/Variant	Primer sequence (5’ to 3’)	Anneal
Primers used for variant validation		
ERO1B	CGAGACTCGACTATGAACTAATCT	57°C
NM_019891.4:c.662C>T	GGTGGGGAGGGGAGTATTTG	
Deletion	CTCTACCAAGCCAATGACCA	65°C
NC_000023.10:g.118373226_118500408del	ATGTAATCTGCCCACCTTGG	
Deletion Left breakpoint	CTCTACCAAGCCAATGACCA	57°C
	AATGTGTTGTTTCCCCCAAG	
Deletion Right breakpoint	AAGGGAGAGGCCATGAGAGT	57°C
	TGTTAAGATTTGACAAAGATGGGC	
RYR2	TGGGTGACAGAGTGAGATGC	60°C
NM_001035.3:c.1893insC	CGGCAAATCCATATTTTGCT	
NAALADL2	AGATCACGCCACTGCACTC	61°C +
NM_207015.3:c.1534-11_1534-10delinsA	AGAAAAGGTCGGTTTTGCAT	Q
Inversion	CCTGTCATCCCAGCACTTTG	62°C +
NC_000001.10:g.237566103_237566207inv	TCTCCCACCCTTGTCCTTTC	Q
Distal duplication	TCACCAACCAACACACCTTTG	
NC_000001.10:240116680_240116681ins	TATGGCTCCAGGTTCCCTGT	61°C
[GTGTGTGAG;240116016_240116379inv;CCAG]		
Tandem duplication	CAGGCAGTGGGTTAGGTTCA	
NC_000003.11:g.176236711_176237056dup	AATGCTCATCATCACGCCCA	63°C

‘+ Q’ addition of Q-solution (Qiagen)

Primers used for zebrafish RT-PCR when assessing *pgrmc1* expression and *pgrmc1*_MO2 splicing capacity.

*pgrmc1*_Exon1_Forward	CGCTGCCCAAACTCAAGAAAA	57°C
*pgrmc1*_Intron1_Forward	ACTAGTAACACGCAACCTACAG	57°C
*pgrmc1*_Exon2/3_Reverse	CGTACTTCTGTGTGAACTGGG	57°C

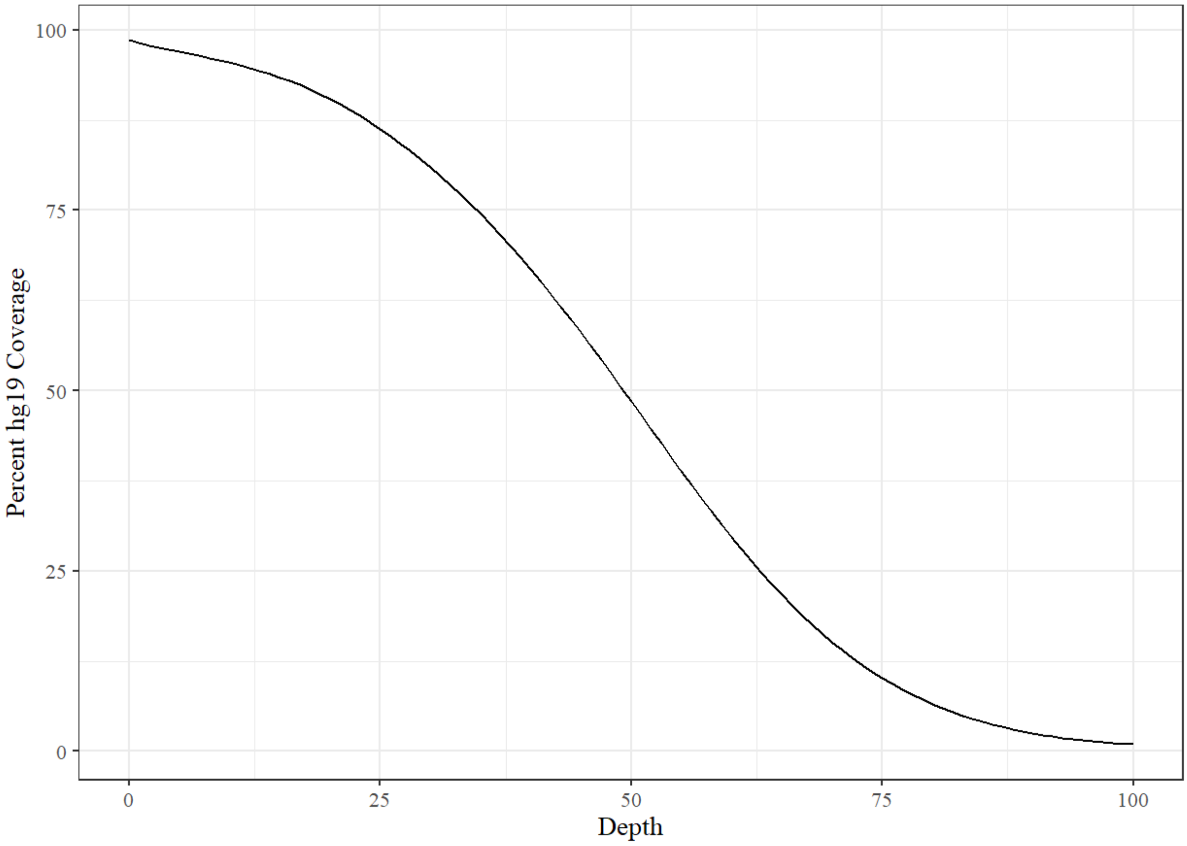

Figure S1 Sequencing coverage plot

Curve shows the cumulative percentage of the target genome (hg19) covered to a minimum sequence depth. Coverage data indicate that at least 80% of the target genome was covered with a minimum of 30 reads.

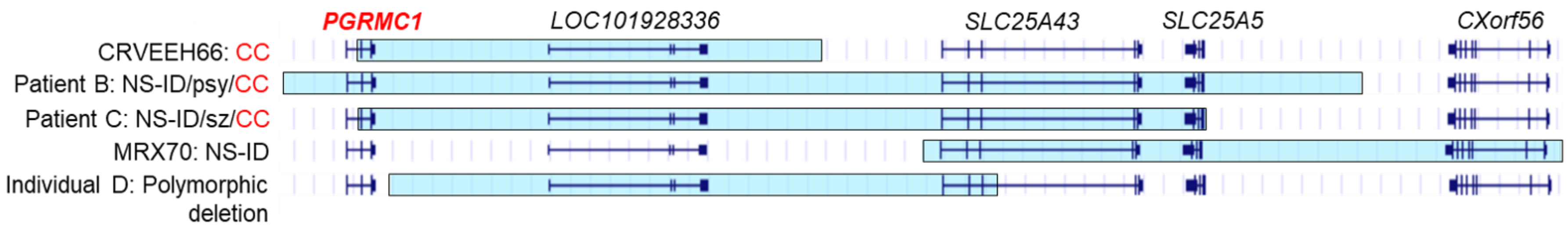

Figure S2 CRVEEH66 deletion in comparison to previously reported deletions

A diagrammatic representation of the deletion observed in family CRVEEH66 compared to individuals reported in a genetic study of non-syndromic intellectual disability by Vandewalle *et al.*(1). Genomic region at Xq24 adapted from the UCSC genome browser (https://genome.ucsc.edu/index.html) with deletions shown in blue. All individuals displayed are male. CRVEEH66, patient B and patient C all have deletions disrupting *PGRMC1* and all have congenital cataracts (CC) as a phenotype. Patient B, patient C and MRX70 all have deletions incorporating *SLC25A5* which was identified at the cause of the non-syndromic intellectual disability (NS-ID). Individual D was reported to have a polymorphic deletion that was also observed in healthy male relatives. psy psychomotor instability, sz seizures. Genomic coordinates of each deletion (hg19, NC_000023.10); CRVEEH66 chrX:g.118373226_118500408del (127kb), patient B chrX:g.118353178_118648846del (295kb), patient C chrX:g.118373287_118606088del (233kb), MRX70 chrX:g.118528138_118705594del (277kb), and Individual D chrX:g.118382049_118548570del (166kb).

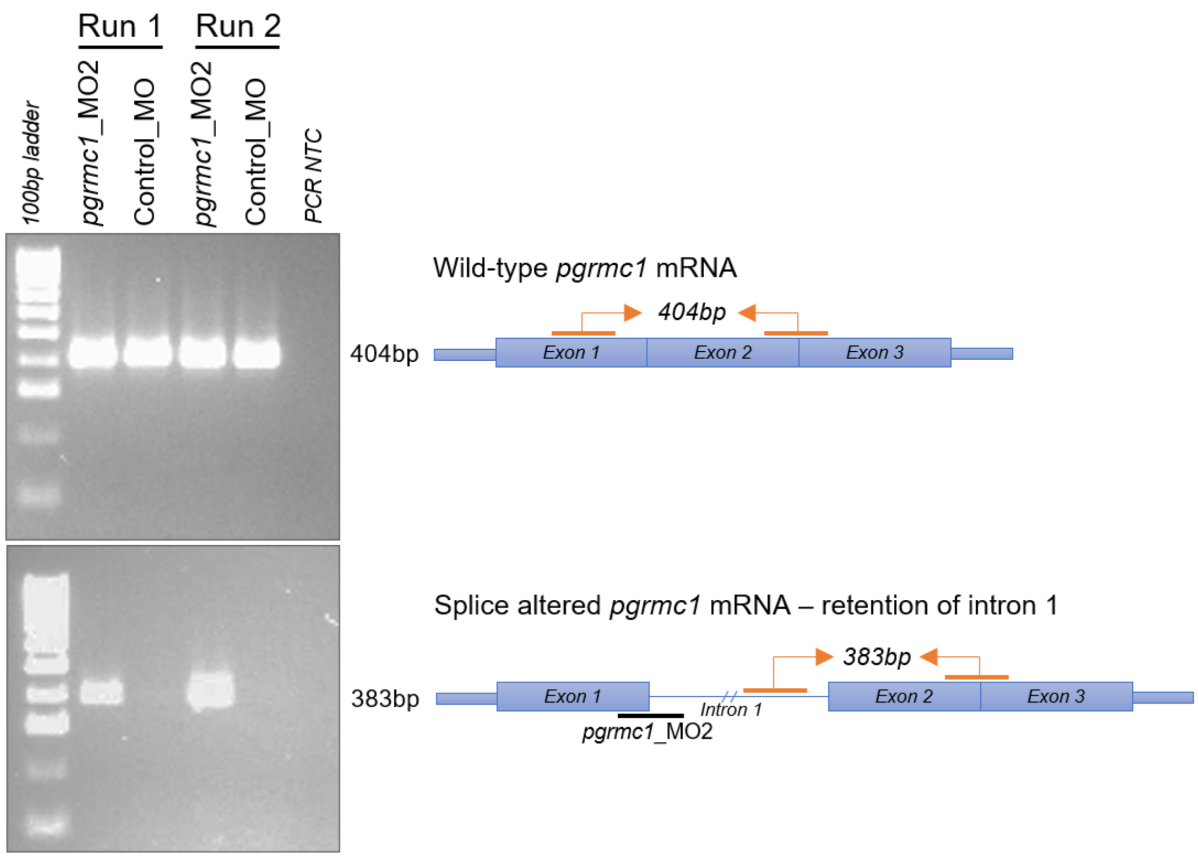

Figure S3 RT-PCR assessment of *pgrmc1*_MO2 splicing capacity

The *pgrmc1*_MO2 and control injected zebrafish larvae, following imaging at 4dpf, were collected for RNA. RT-PCR gel electrophoresis results are displayed with diagrammatic representation of the transcript and primer locations indicated by the orange arrows. The retention of intron 1 (383bp RT-PCR amplicon), within the *pgrmc1* transcript, is observed only in groups treated with *pgrmc1*_MO2. Both control and *pgrmc1*_MO2 groups displayed normally processed *pgrmc1* (404bp RT-PCR amplicon) that would have been translated into functional protein. The *pgrmc1*_MO2 experimental group was expected to display two bands when testing for wild-type *pgrmc1* transcript. The second larger (approx. 1.2kb) band was not observed, either due to low quantity and/or PCR bias. Collectively, this indicates that *pgrmc1*_MO2 efficiency is not optimal. All RT-PCR products were confirmed using Sanger sequencing.

Supplemental Reference

1 Vandewalle, J., Bauters, M., Van Esch, H., Belet, S., Verbeeck, J., Fieremans, N., Holvoet, M., Vento, J., Spreiz, A., Kotzot, D. *et al.* (2013) The mitochondrial solute carrier SLC25A5 at Xq24 is a novel candidate gene for non-syndromic intellectual disability. *Human genetics*, **132**, 1177-1185.
